# Tracking KLRC2 (NKG2C)+ memory-like NK cells in SIV+ and rhCMV+ rhesus macaques

**DOI:** 10.1371/journal.ppat.1007104

**Published:** 2018-05-31

**Authors:** Daniel R. Ram, Cordelia Manickam, Brady Hueber, Hannah L. Itell, Sallie R. Permar, Valerie Varner, R. Keith Reeves

**Affiliations:** 1 Center for Virology and Vaccine Research (CVVR), Beth Israel Deaconess Medical Center/Harvard Medical School, Boston, Massachusetts, United States; 2 Human Vaccine Institute, Duke University Medical Center, Durham, North Carolina, United States; 3 Ragon Institute of Massachusetts General Hospital, MIT and Harvard, Cambridge, Massachusetts, United States; Emory University, UNITED STATES

## Abstract

Natural killer (NK) cells classically typify the nonspecific effector arm of the innate immune system, but have recently been shown to possess memory-like properties against multiple viral infections, most notably CMV. Expression of the activating receptor NKG2C is elevated on human NK cells in response to infection with CMV as well as HIV, and may delineate cells with memory and memory-like functions. A better understanding of how NKG2C+ NK cells specifically respond to these pathogens could be significantly advanced using nonhuman primate (NHP) models but, to date, it has not been possible to distinguish NKG2C from its inhibitory counterpart, NKG2A, in NHP because of unfaithful antibody cross-reactivity. Using novel RNA-based flow cytometry, we identify for the first time true memory NKG2C+ NK cells in NHP by gene expression (KLRC2), and show that these cells have elevated frequencies and diversify their functional repertoire specifically in response to rhCMV and SIV infections.

## Author summary

Natural killer (NK) cells are a crucial component of the early innate immune response, and although NK cell responses have been thought be only non-specific, recent evidence suggests that NK cells are capable of expanding with some specificity, indicative of a memory-like adaptive response. The activating receptor NKG2C has been one cell surface protein associated with this memory-like NK cell expansion in the context of CMV and HIV infection in humans, yet very little is known about NKG2C+ NK cells in non-human primate (NHP) animal models. This is predominantly because there are no antibodies that can distinguish NKG2C from other NKG2 family molecules in NHP. Because vaccine and cure-related studies for HIV rely heavily on NHP models, this is a significant impediment towards understanding an NK cell population that may possibly improve responses to HIV. In this paper we present a solution, by adapting a technique whereby mRNA specific to NKG2C and NKG2A (KLRC2 and KLRC1, respectively) is fluorescently labeled while the cell is simultaneously stained using traditional flow cytometry, and provide a first-ever characterization of NKG2C+ NK cells in NHP. Further, we show that NKG2C+ NK cells expand in a memory-like fashion following rhCMV and SIV infections.

## Introduction

Although NK cells have traditionally been thought to be innate immune cells that lack the antigen-specificity seen in the adaptive immune system, NK cells have very recently been reported to possess memory and memory-like functions [1–8]. Though this area of investigation is currently developing, subpopulations of NK cells that express NKG2C (CD159C) in humans or Ly49H and Ly49P in mice mobilize in response to CMV infection [9–13]. While this phenomenon has been described in human and murine studies, because of technical limitations it has not yet been possible to examine memory and memory-like NKG2C+ NK cells in NHP models. This is predominantly attributed to the high degree of homology in NHP between the extracellular domains of two NKG2 isoforms, activating NKG2C and inhibitory NKG2A –making the two indistinguishable via currently available antibodies and standard measurements [14, 15]. NHP models are crucial to multiple areas of medical research, including HIV and CMV infectious disease study and transplant biology [16–18] since the murine system does not always approximate human immunology. As such, the inability to study NKG2C+ memory NK cells in NHP models remains a major research deficit.

NKG2C and NKG2A both belong to the C-type lectin family of NK cell receptors. NKG2C recruits the adaptor protein DAP12, which has an ITAM (immunoreceptor tyrosine-based activation motif), and NKG2A has two ITIM (immunoreceptor tyrosine-based inhibitory motif) domains, which lead to recruitment of phosphatases, and downregulation of signaling [19, 20]. Because these two proteins act in opposition to each other, it is crucial to discriminate between cells that express either protein in order to more accurately determine what role these cells play during infection. As a result, we aimed to utilize RNA hybridization technology recently adapted for flow cytometry (PrimeFlow) to label the gene transcripts of rhesus macaque NKG2A and NKG2C (KLRC1 and KLRC2, respectively), taking advantage of several nucleotide differences between the two transcripts, in order to distinguish cells that transcribed these isoforms. This approach should allow simultaneous detection of surface and intracellular proteins as well as gene transcript levels with a single-cell resolution using polychromatic flow cytometry. In addition to differentiating between KLRC1+ and KLRC2+ NK cells, this technology should allow evaluation of NK cell population diversity, including memory cells, in the context of “normal” CMV infection, in chronic SIV infection, and in CMV-negative specific pathogen free (SPF) rhesus macaques. Understanding how KLRC1±KLRC2± NK cells function in the context of infection will help improve our basic understanding of NK cell biology, potentially inform preclinical HIV vaccine or cure studies relying on macaque models, and provide a significant technological advance to the study of memory NK cells in primates.

## Materials and methods

### Ethics statement

All animals were housed at the Tulane Primate Research Center (TNPRC) or Biomere (Worcester, MA). All study blood samplings were reviewed and approved by the Tulane University Institutional Animal Care and Use Committee or the Biomere Institutional Animal Care and Use Committee under protocol numbers 16–08 and 17–02. All animal housing and studies were carried out in accordance with recommendations detailed in the Guide for the Care and Use of Laboratory Animals of the National Institutes of Health with recommendations of the Weatherall report; “The use of non-human primates in research”. AAALAC numbers for TNPRC and Biomere– 00594 and 1152, respectively. Animals were fed standard monkey chow diet supplemented daily with fruit and vegetables and water ad libitum. Social enrichment was delivered and overseen by veterinary staff and overall animal health was monitored daily. Animals showing significant signs of weight loss, disease or distress were evaluated clinically and then provided dietary supplementation, analgesics and/or therapeutics as necessary. No animals were euthanized as part of this research.

### Animals

Thirty Indian rhesus macaques were analyzed in this study: ten specific pathogen free (SPF) animals (rhCMV- and SIV-negative), twelve otherwise naïve animals that were naturally infected with rhCMV (rhCMV+), and eight chronically infected with SIV_mac251_ (all of which rhCMV+). SPF animals and age-matched non-SPF/rhCMV+ macaques were housed at the Tulane National Primate Research Center (TNPRC). SIV-infected macaques and additional rhCMV+ animals were housed at Biomere. All animals were colony housed until on study and then infected animals were housed under BSL2 conditions.

### Blood processing

Whole blood was collected into EDTA-treated tubes. Peripheral blood mononuclear cells (PBMCs) were isolated by density-gradient centrifugation layered over 100% Ficoll. Cell aliquots were immediately analyzed or cryopreserved in 90% FBS, 10% DMSO (Sigma) and stored in liquid nitrogen vapor.

### RNA-Flow

PBMCs were thawed and rested for 12h in R10 media at 37°C prior to surface and intracellular staining followed by RNA-Flow hybridization using the manufacturer’s recommended protocol (PrimeFlow, Affymetrix, Santa Clara, CA) with the antibodies detailed in the flow cytometry section below, and with rhesus macaque-specific KLRC1 and KLRC2 probesets. Rhesus-specific probesets were custom designed with the assistance of Affymetrix (Santa Clara, CA) specifically for this project to target rhesus KLRC1 and KLRC2. Target probes sequences for KLRC1 and KLRC2 are shown in **S1 and S2 Figs**, as are ‘blocking probe’ sequences used to prevent nonspecific binding. The blocking probes were designed in order to avoid amplifying/detecting undesired NKG2 homologues. Blocking probes do not have the ability to form branched DNA structures which hybridize to the label probe fluorophores as opposed to the target probes which are able to hybridize to label probe fluorophores. Both target probes and blocking probes were simultaneously added at the target probe hybridization step as per the manufacturer’s protocol. Probesets were labeled with Alexa-488 (KLRC1) and Alexa-647 (KLRC2) fluorophores by Affymetrix (Santa Clara, CA). All KLRC1 and KLRC2 gates were determined for each sample by comparing the samples stained with all antibodies and probesets with samples only stained with antibodies (no probeset control).

### Flow cytometry

All antibodies used were purchased from BD Biosciences unless specified otherwise. For phenotypic panels antibodies against the following cell antigens were used: CD2 (RPA2.10), CD3 (SP34.2), CD337 (p30-15), CD14 (MϕP9), CD20 (L27), CD16 (3G8), CD56 (NCAM16.2), HLA-DR (G46-6), CD8α (SK1), KIR2D (NKVFS, Miltenyi [this antibody recognizes KIR3D in NHP as shown by Pomplun, N. et al. [21]]), CD159a/c (Z199, Beckman Coulter), CD366 (F38-2E2, Biolegend). Additionally, antibodies used for functional assays included TNF-α (MAb11), IFN-γ (B27), CD107a (H4A3). Flow cytometry data was acquired on a LSRII (BD Biosciences, La Jolla, CA) and analyzed with FlowJo software (version 10.2, Tree Star, Ashland, OR). *t*-SNE (*t*-distributed stochastic neighbor embedding) was carried out using the *t*-SNE feature in FlowJo using 1000 iterations and a perplexity of 20.

### Functional assays

PBMCs from animals were prepared at 37°C and cells were cultured with Golgi Plug and Golgi Stop (BD Biosciences, concentrations as recommended by manufacturer), and PMA (3.3μg/mL, Sigma) and Ionomycin (5μg/mL, Sigma) or with unlabeled anti-CD16 (3G8, 20μg/mL) and cross-linked with F(ab’)_2_ (20μg/mL, Jackson Immunoreserach, West Grove, PA) for 14h in R10 media (RPMI + 10% FBS + 2% PenStrep (Gibco)).

### rhCMV UCD52 whole-virion ELISA

Rhesus plasma was assessed for rhCMV-specific IgG by a previously reported rhCMV UCD52 whole virion ELISA [22]. After plates were coated with 4,400 PFU/mL of rhCMV UCD52 virus, the previously reported procedure was followed. The positivity threshold for detectable antibody levels was set to equal twice the OD of a rhCMV-seronegative plasma control at the starting dilution (1:30).

### Statistical analyses

Statistical and graphing analyses were performed with GraphPad Prism 7.0 software (GraphPad Software, La Jolla, CA). Nonparametric Mann-Whitney *U* tests and Wilcoxon tests were used where indicated, and a *p-*value of *p* < 0.05 was considered to be statistically significant.

## Results and discussion

### Identification of KLRC1+ and KLRC2+ NK cells in rhesus macaques

**Fig 1:**
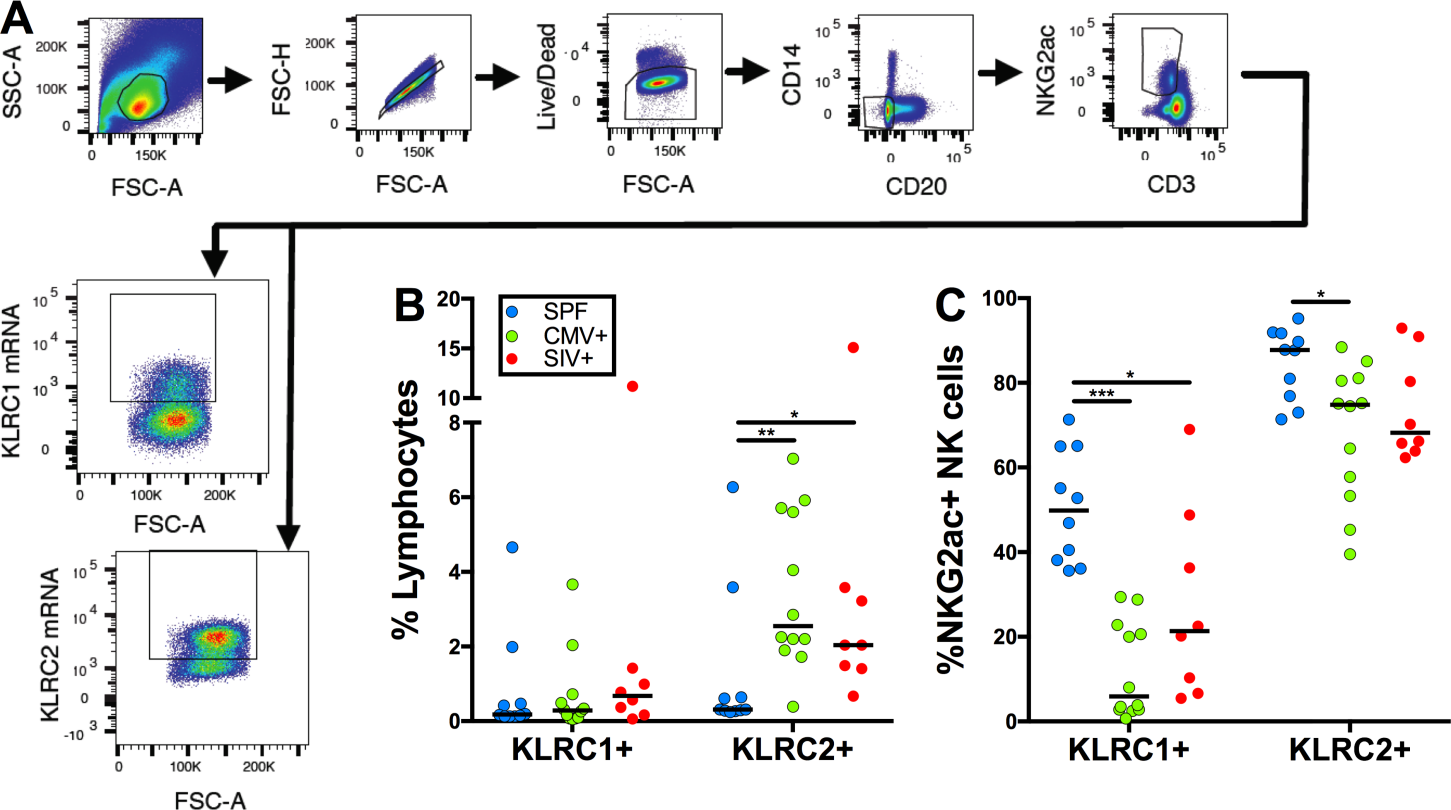
KLRC2 mRNA levels are elevated in CMV and SIV infection. (A) Representative gating strategy showing the criteria for identifying NK cells, as well as strategy for differentiating KLRC1+ or KLRC2+ populations. (B) Data showing KLRC1+ or KLRC2+ NK cells as a percentage of all lymphocytes, and (C) as a percentage of NKG2AC+ NK cells. The horizontal bars in (B) and (C) indicate medians. Each point corresponds to a single animal: SPF (n = 10), CMV (n = 12) and SIV (n = 8). Mann-Whitney *U* test; *p < 0.05, **p < 0.01, ***p < 0.0001.

Total NK cells were identified among PBMC in rhesus macaques using traditional phenotypes optimized by our laboratory [5, 23–27]: CD3-CD14-CD20-NKG2A+ (**Fig 1A**). Unsurprisingly, the anti-NKG2A antibody was unable to distinguish between NKG2A and NKG2C, as has been previously shown by the Letvin lab whereby they showed that anti-human NKG2A antibodies were cross-reactive with four NKG2C alleles [14]. As a result we now classify bulk cells that are positive for this antibody as NKG2AC+ NK cells. Using RNA-Flow (see Methods) we next identified within NKG2AC+ NK cell populations those cells that expressed transcripts of the genes coding for NKG2A and NKG2C (KLRC1 and KLRC2, respectively), and accurately quantified the frequency of NK cells expressing one or both of these genes (**Fig 1A–1C**). Interestingly, absolute frequencies of both KLRC1+ and KLRC2+ NK cells (**Fig 1B**) were lower in SPF animals compared to either rhCMV+ or SIV-infected macaques—(KLRC1) 0.18%, 0.29%, and 0.68% of lymphocytes in SPF, rhCMV+, and SIV+ animals respectively; and (KLRC2) 0.31%, 2.55%, and 2.04% of lymphocytes in SPF, rhCMV+, and SIV+ animals respectively. These data demonstrate that while NK cells are less frequent in SPF animals in general, following rhCMV infection a less than 2-fold non-significant increase occurs in KLRC1+ NK cells, but the increase in KLRC2+ NK cells is 12-fold. This dramatic observation is congruent with other findings in human research, which show elevation of NKG2C+ NK cells specifically following CMV infection [9–11, 28]. Examining the frequency of KLRC1+ and KLRC2+ NK cells relative to the total NK cells population (**Fig 1C**) also revealed that in rhCMV+ and SIV-infected macaques there was an obvious reduction in KLRC1+ NK cells in lieu of KLRC2+ NK cells, but surprisingly, expression of both KLRC1 and KLRC2 remained high in NK cells from SPF animals.

### rhCMV infection favors an increased frequency of KLRC2 (NKG2C)+ activated NK cells

**Fig 2:**
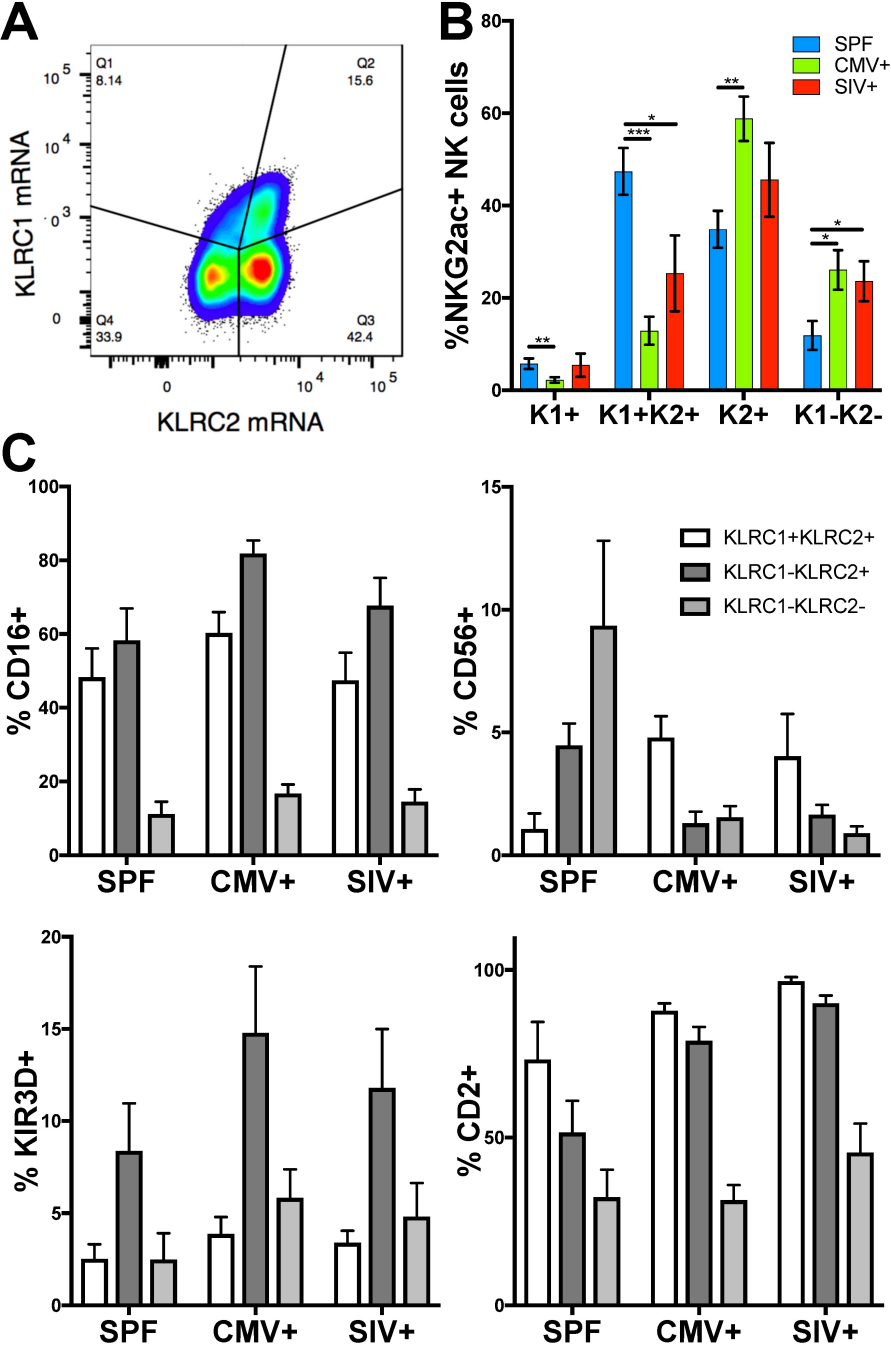
NK cell phenotyping reveals quadrant-specific distribution and phenotypic differences among animal groups. (A) Representative gating strategy showing identification of KLRC1±KLRC2± NK cells among NKG2AC+ NK cells as shown in Fig 1A. (B) Distribution of NK cell KLRC1±KLRC2± subpopulations in SPF and infected animals. K1+, K1+K2+, K2+ and K1-K2- correspond to KLRC1+KLRC2-, KLRC1+KLRC2+, KLRC1-KLRC2+ and KLRC1-KLRC2- populations, respectively. (C) Phenotypic markers on KLRC1±KLRC2± NK cells in SPF and infected animals. Data are show as mean ± SEM. The numbers of animals shown per group are as follows: SPF (n = 10), CMV (n = 12) and SIV (n = 8). Mann-Whitney *U* test was used to compare quadrant populations of different infection groups; Wilcoxon test was used to compare different quadrant populations within the same infection group; *p < 0.05, **p < 0.01, ***p < 0.0001 for panel B. Statistical data for panel C is presented in S1 Table.

**Fig 3:**
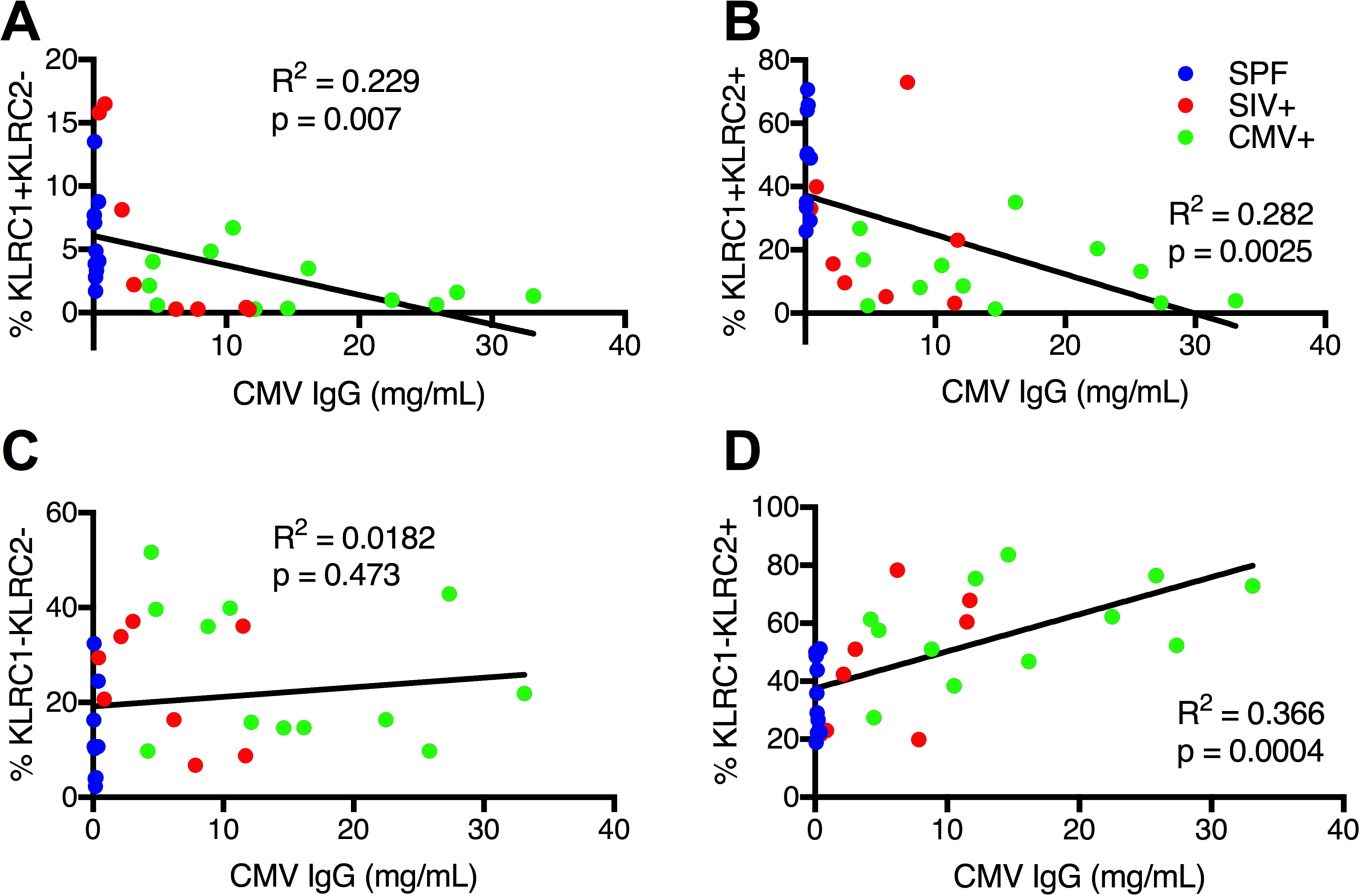
Increased frequencies of KLRC1-KLRC2+ NK cells are positively correlated with higher rhCMV-specific IgG. Linear regression analysis showing correlation between rhCMV-binding antibody equivalents and (A)KLRC1+KLRC2-, (B)KLRC1+KLRC2+, (C) KLRC1-KLRC2- and (D) KLRC1-KLRC2+ NK cells.

To further clarify our findings, we re-optimized our technical approach to measure both KLRC1 and KLRC2 simultaneously. Using the gating strategy shown in **Fig 2A** we were able to clearly distinguish four distinct NK cell populations by expression of KLRC1 and KLRC2. Strikingly, this analysis revealed that in the absence of rhCMV infection, a KLRC1+KLRC2+ population is dominant (**Fig 2B**). In contrast, in rhCMV+ animals (rhCMV+ and SIV+ groups) the predominant population was single-positive KLRC1-KLRC2+. Consistently, KLRC1+KLRC2- and KLRC1-KLRC2- represented minority populations among all animal groups and could represent precursor or aberrant NK cells outside the normal NK cell repertoire. While the presence of the KLRC1-KLRC2- population was surprising, it must be noted that the Z199 clone that detects human NKG2A and rhesus macaque NKG2A and NKG2C is promiscuous and could be identifying minor NKG2 isoforms[14]. The extreme specificity and blocking probes used in the RNA-based flow cytometric technique make it highly unlikely to have non-specific signals (**S1 and S2 Figs**). The presence of a double-negative population is more likely resulting from some samples where mRNA levels being below the threshold of detection of this assay. Regardless, these findings point to the overall importance of this study which are now able to confirm true KLRC2+ (NKG2C) NK cells in macaques which have only been incompletely described previously. Also, consistent with observations in humans we also find that both KLRC1 and KLRC2 are expressed on minor populations of T cells (**S3 Fig).** Finally, we can determine that the observation that rhCMV+ and SIV+ animals have higher relative and absolute frequencies of KLRC1-KLRC2+ compared to KLRC1+KLRC2+ NK cells is likely analogous to the memory and memory-like functions observed in human CMV infection [10, 12, 13], whereby prior to CMV exposure both inhibitory NKG2A and activating NKG2C are expressed, but NKG2A is downregulated following infection. Further supporting the notion that CMV specifically expands NKG2C+ NK cells, we found a significant positive correlation between increasing KLRC1-KLRC2 (NKG2C)+ NK cells and rhCMV-binding IgG, as a surrogate indicator of virus replication (**Fig 3D**). Concurrently there was a significant negative correlation between frequencies of KLRC1+KLRC2± NK cells and increasing rhCMV-specific IgG (**Fig 3A and 3B**). There was, however, no association between rhCMV-specific IgG and KLRC1-KLRC2- NK cells (**Fig 3C**). Interestingly, no correlation was found between SIV viral loads (median 3.00 x 10^6^ virus copies/ml; range 5.66 x 10^4^ to 3.30 x 10^7^) and any of the NK cell subpopulations. Collectively, these data suggest that perturbations of KLRC1±KLRC2± NK cells is primarily driven by rhCMV status.

We next wanted to confirm phenotypically that the KLRC1 and KLRC2 definitions were indeed identifying NK cell subpopulations that are analogous to their human counterparts in which NKG2C+ NK cells are more activated and differentiated. Indeed FcRγIII receptor CD16 was higher in rhCMV+ and SIV-infected animals compared to SPF and was consistently higher on KLRC2+ NK cells. (**Fig 2C, S1 Table**). CD56 is typically expressed on most circulating NK cells in humans, but is expressed on only a small frequency of cytokine-producing or less differentiated NK cells in macaques [29]. Consistent with this notion, we found CD56 expression was higher in general on homeostatic KLRC1+KLRC2+ NK cells, but was poorly expressed on KLRC1-KLRC2+ NK cells expanded by rhCMV infection seen in the CMV+ and SIV+ groups. While this trend was not present in SPF animals we noted that expression of CD56 was generally higher in SPF samples as compared to the CMV+ and SIV+ groups, though it was not statistically significant. In general KIR expression is increased as NK cells differentiate and it was hypothesized that rhCMV or SIV infection are increasing activation and differentiation. Indeed expression of the common macaque KIR, KIR3D, was lower in all SPF animals and had the highest expression on KLRC1-KLRC2+ NK cells. CD2, which has been shown to synergize with NKG2C to promote adaptive NK cell functions [30], was also found to be increased on KLRC2+ cells in rhCMV+ macaques. Unfortunately, cross-reactive antibodies against the CD57 carbohydrate epitope, also associated with memory NK cell phenotypes, do not currently exist for monkeys and thus could not also be evaluated here. Nonetheless, these findings collectively suggest this population is generally more activated and differentiated and has a phenotype consistent with adaptive functions.

### KLRC2+ NK cell diversity is dependent on infection status

**Fig 4:**
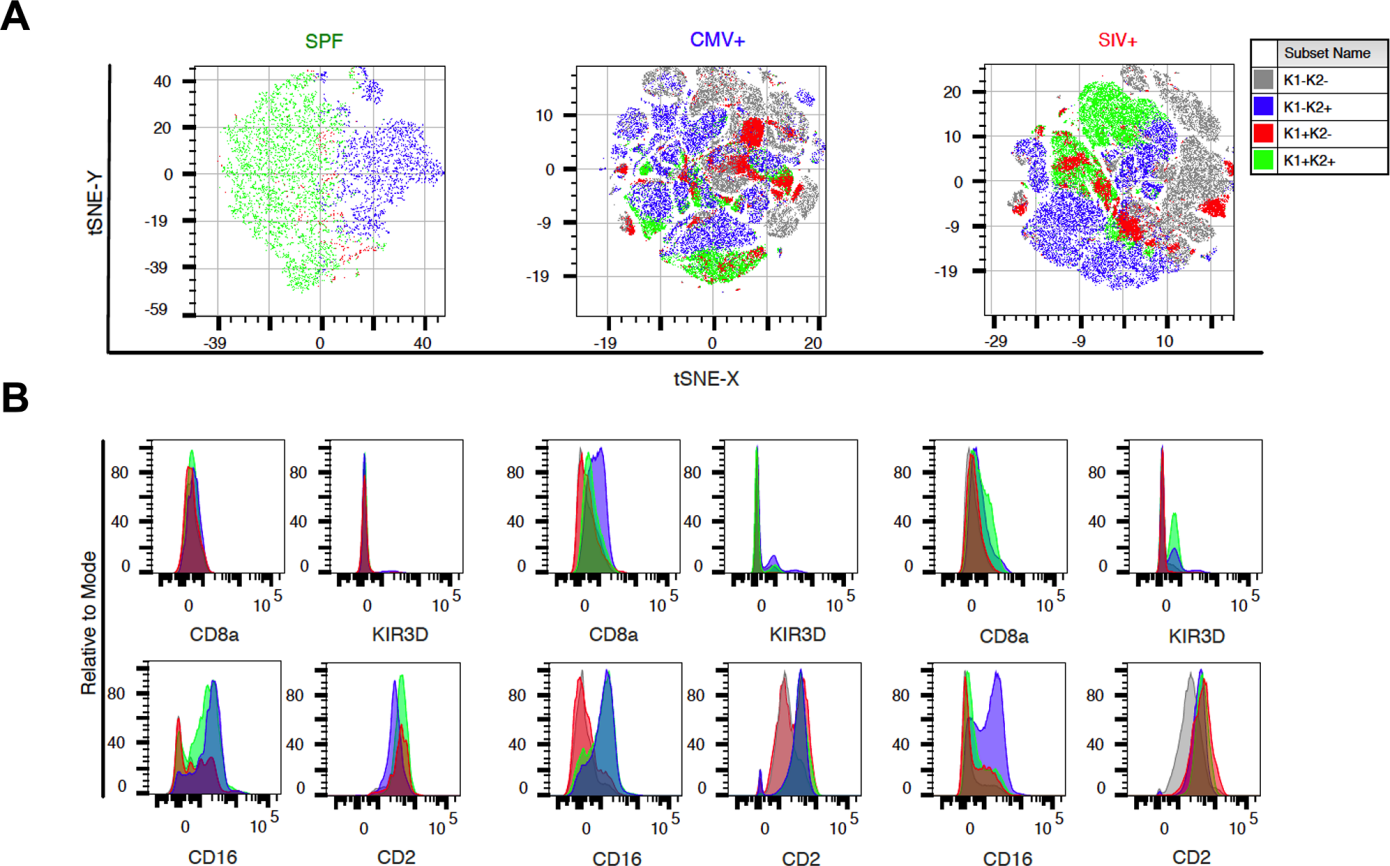
Infection increases NK cell diversity. (A) *t*-SNE plots showing relationship among KLRC1±KLRC2± NK cells within SPF (n = 10), SIV+ (n = 8) or CMV+ (n = 12) animals. (B) Phenotypic histograms per group on which *t*-SNE plots were generated in (A). Histogram colors match their corresponding populations.

Since our analyses suggested rhCMV infection may be driving KLRC2+ NK cell expansion, we next used *t*-SNE to evaluate NK cell subpopulation clustering and diversity. NK cells from SPF animals clustered into two major groups–corresponding with KLRC1+KLRC2+ and KLRC1-KLRC2+ populations. In contrast, NK cells from rhCMV+ and SIV+ animals clustered into far more minor and distinct groups (**Fig 4A**). The phenotypic characteristics of these groups were also highly variable depending on infection status and subpopulation (**Fig 4B**). These data suggest that there is a greater diversity of NK cell subpopulations following infection with rhCMV or SIV as compared with the uninfected SPF group. These findings are in strong agreement with previous analyses by showing that human NK cell diversity increases following infection with HIV and other pathogens [31–33].

### NK cell functional characteristics are preserved among subpopulations

**Fig 5:**
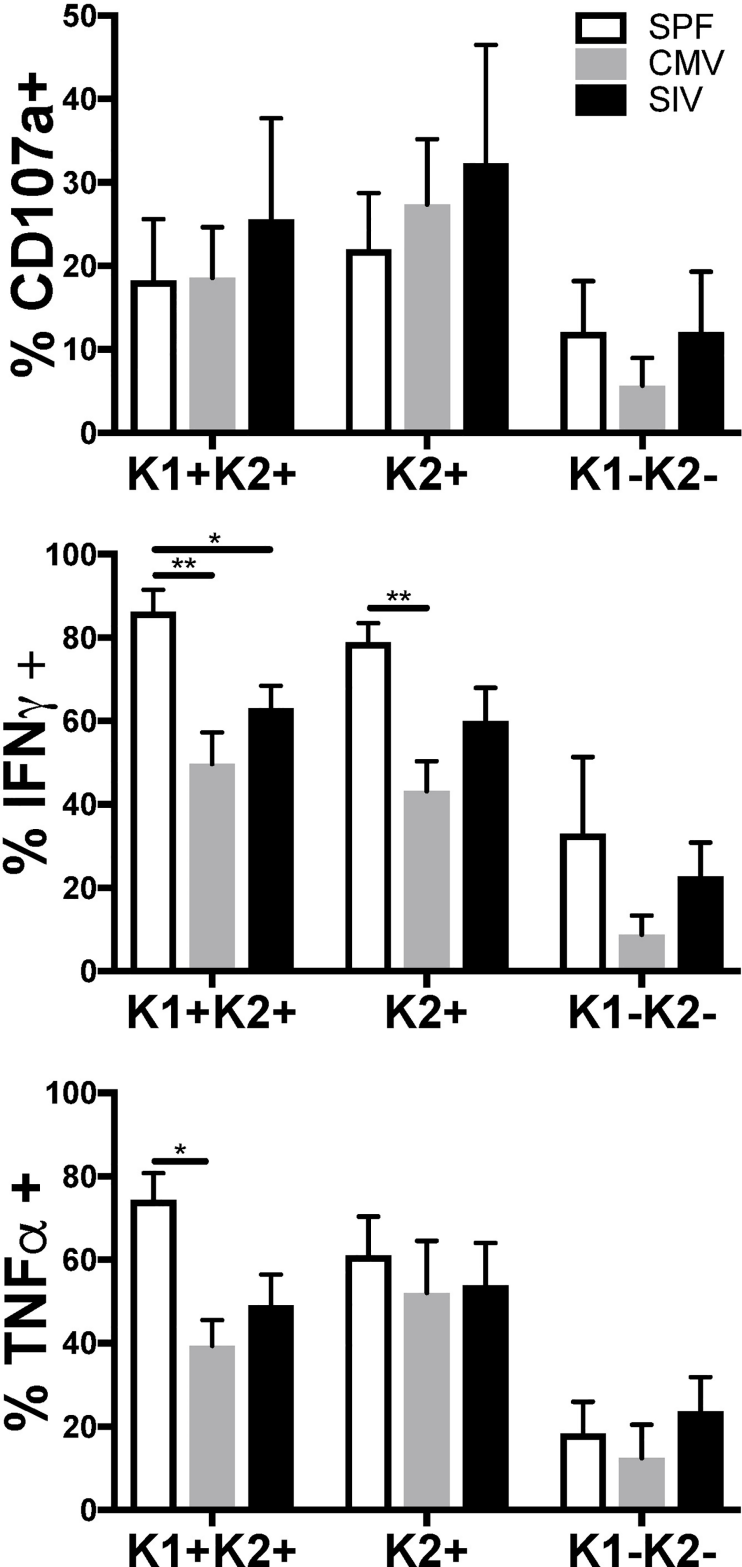
KLRC2+ NK cells are functionally responsive. Data showing CD107a expression, or production of IFN-γ and TNF-α following stimulation with PMA+ ionomycin in NK cell subpopulations from SPF, CMV+ or SIV+ animals. Means + SEM are shown. Numbers of animals per independent experiment: SPF (n = 5), CMV (n = 5) and SIV (n = 5). Mann-Whitney *U* test; *p < 0.05, **p < 0.01, ***p < 0.0001.

Next we wanted to examine whether there were any functional differences among each of the KLRC1+ and KLRC2+ populations. Mitogen stimulation revealed that all NK cell subsets from all animal groups were capable of surrogate cytotoxic (CD107a) and cytokine-based (IFN-γ, TNF-α) responses (**Fig 5**). Unfortunately, the number of positive events for the KLRC1+KLRC2- population were too few for us to reliably report from the functional assay. Nevertheless, the remaining quadrant populations were functional, with the KLRC1+KLRC2+ and KLRC1-KLRC2+ populations demonstrating the most robust responses. Interestingly, upon mitogen stimulation we observed that NK cells from SPF animals produced proportionately greater cytokines, which could be indicative of a more immature status as expected given the lack of virus exposure. Following stimulation through CD16 that could mimic ADCC, again all NK cell subpopulations were functionally competent. Perhaps even more obvious in this assay, NK cells from SPF animals favored cytokine production, whereas those from rhCMV+ animals were more adept for CD107a upregulation as a surrogate indicator of cytotoxicity (**S4 Fig**). These findings further suggest that CMV infection is necessary to activate and prime cytotoxic functions, particularly those dependent on antibodies and corroborates findings in humans mediated by NKG2C+ γ-chain deficient memory-like NK cells [13]. Indeed, the memory-like programming observed for rhCMV could be epigenetic in nature if not memory per se. Collectively, all NK subpopulations from SIV-infected animals were functionally responsive to mitogen, but were poorly responsive to CD16 cross-linking. These findings are well in-line with previous observations of NK cell dysfunction in HIV and SIV infections. In this paper we present a cross-sectional analysis of several infected and uninfected animals. Further studies need to be carried out in order to examine the kinetics of infection and how SIV or CMV may play a role in shaping the NK cell repertoire. Importantly, we also show that with this technique we are able to also examine and identify KLRC1 and KLRC2+ NK cells from peripheral lymphoid tissues such as the spleen and primary sites of infection such as the colon (**S5 Fig**). This will allow us to examine the role that these NK cell populations play in the earliest stages following infection in the relevant tissues.

In conclusion, we report that it is now possible to specifically identify NKG2C+ and NKG2A+ macaque NK cells using their respective transcripts, KLRC2 and KLRC1, as proxy. Further, we show for the first time that rhCMV infection results in increased NK cell diversity and a specific increase in NKG2C+ NK cells. Altogether these findings strengthen the argument for NKG2C+ memory and memory-like NK cells arising in response to CMV and lentivirus infections and provide a tangible NHP model in which to study them.

## Supporting information

S1 Fig KLRC1 and KLRC2 probesets are specific to their respective genes. (A) Graphical representation of KLRC1 (red) and KLRC2 (purple) genes, and the regions covered by KLRC1 probeset (blue) and KLRC2 probeset (yellow). The cyan blocks labeled “B” are the positions of the blocking probes. (B) mRNA sequences for KLRC2 and KLRC1 showing the areas targeted by the probesets and the blocking probes. (C) Table showing probeset and blocking probe sequences for both KLRC2 and KLRC1. The color scheme for (B) and (C) is the same as in (A).
(JPG)

S2 Fig Alignment of KLRC1 and KLRC2. Both genes are aligned to show the sequence-specific location of all probes specific for KLRC1 and KLRC2 of all probes in S1 Fig. KLRC1 gene (red) and KLRC2 gene (purple) genes; KLRC1 probeset (blue) and KLRC2 probeset (yellow); Blocking probes (cyan).
(JPG)

S3 Fig KLRC1 and KLRC2 are expressed at low frequencies on CD3+ T cells. Representative plots showing expression of KLRC1 and KLRC2 on NK cells (CD14-CD20-CD3-NKG2ac+) and CD3+ T cells (CD14-CD20-CD3+NKG2ac±).
(TIF)

S4 Fig KLRC2± NK cells are responsive to CD16 cross-linking. Data showing CD107a expression, or production of IFN-γ and TNF-α following stimulation with anti-CD16 cross-linked with F(ab’)_2_ in NK cell subpopulations from SPF, rhCMV+ or SIV+ animals. Means + SEM are shown. Numbers of animals per independent experiment: SPF (n = 5), CMV (n = 5) and SIV (n = 5). Mann-Whitney *U*; *p < 0.05, **p < 0.01, ***p < 0.0001.
(TIF)

S5 Fig KLRC1±KLRC2± NK cells can be identified in peripheral lymphoid and gut tissue. Representative flow plots showing KLRC1±KLRC2± quadrant populations in (A) Spleen and (C) Colon, as well as a distribution of NK cell KLRC1±KLRC2± subpopulations in CMV and SIV infected animals in (B) Spleen and (D) Colon.
(TIF)

S1 Table NK cell phenotypic *p*-values. Multiple comparisons carried out in the phenotypic assays from **Fig 2C.** Quadrants are represented as K2+ (KLRC1-KLRC2+), K1+K2+ (KLRC1+KLRC2+) and K1-K2- (KLRC1-KLRC2-). Shaded cells indicate comparison events that were deemed significant at *p* < 0.05. Non-parametric Wilcoxon test was used for inter-quadrant comparisons, and the non-parametric Mann-Whitney *U* test was used for inter-infection group comparisons.
(DOCX)
