# Supplementary figures and images for: Tracking KLRC2 (NKG2C)+ memory-like NK cells in SIV+ and rhCMV+ rhesus macaques
Source: PLoS Pathog. 2018 May 31;14(5):e1007104. doi: 10.1371/journal.ppat.1007104 (PMC5997355; doi:10.1371/journal.ppat.1007104)

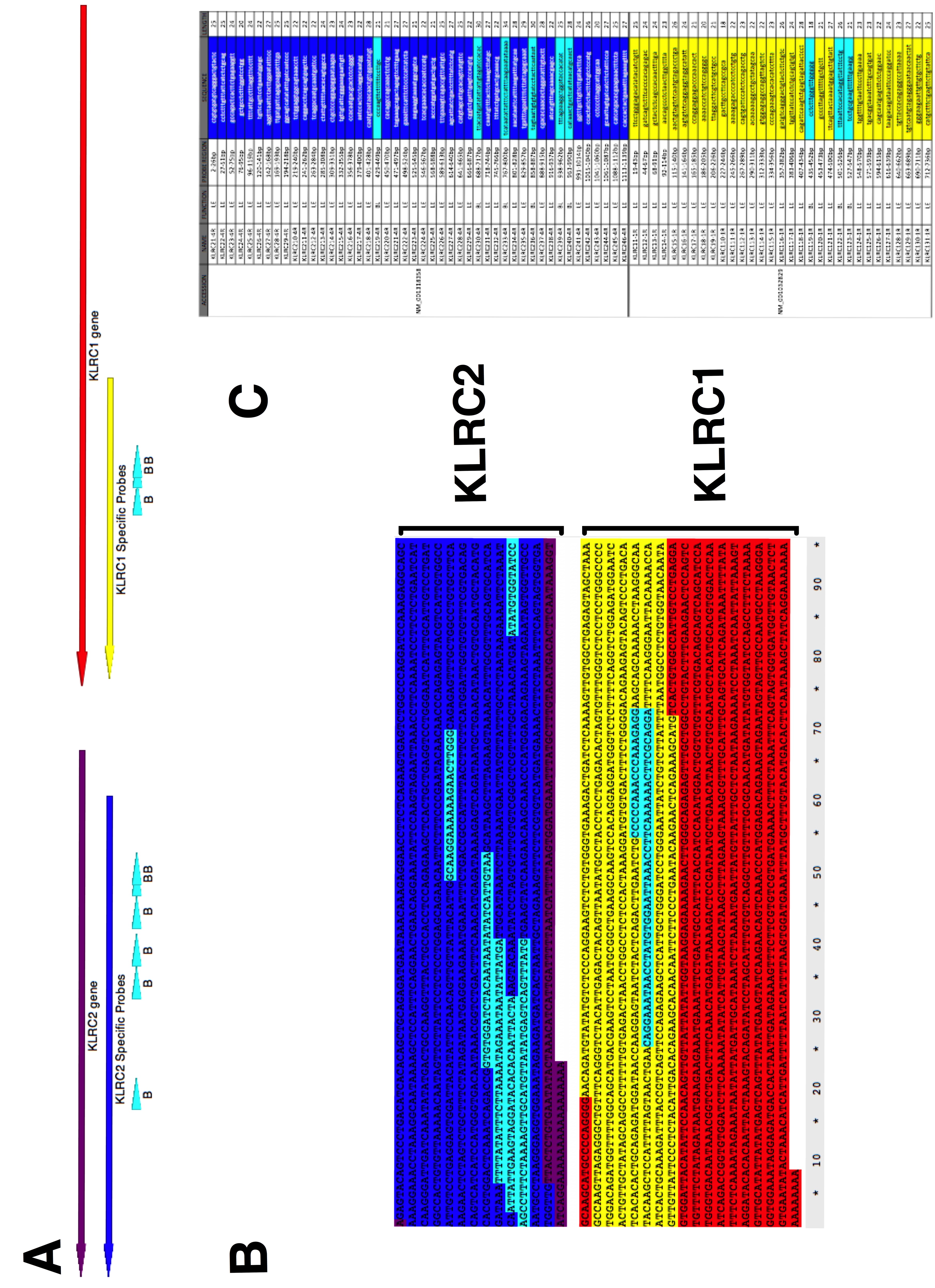

Supplement: S1 Fig — (A) Graphical representation of KLRC1 (red) and KLRC2 (purple) genes, and the regions covered by KLRC1 probeset (blue) and KLRC2 probeset (yellow). The cyan blocks labeled “B” are the positions of the blocking probes. (B) mRNA sequences for KLRC2 and KLRC1 showing the areas targeted by the probesets and the blocking probes. (C) Table showing probeset and blocking probe sequences for both KLRC2 and KLRC1. The color scheme for (B) and (C) is the same as in (A). (JPG) [file ppat.1007104.s001.jpg]

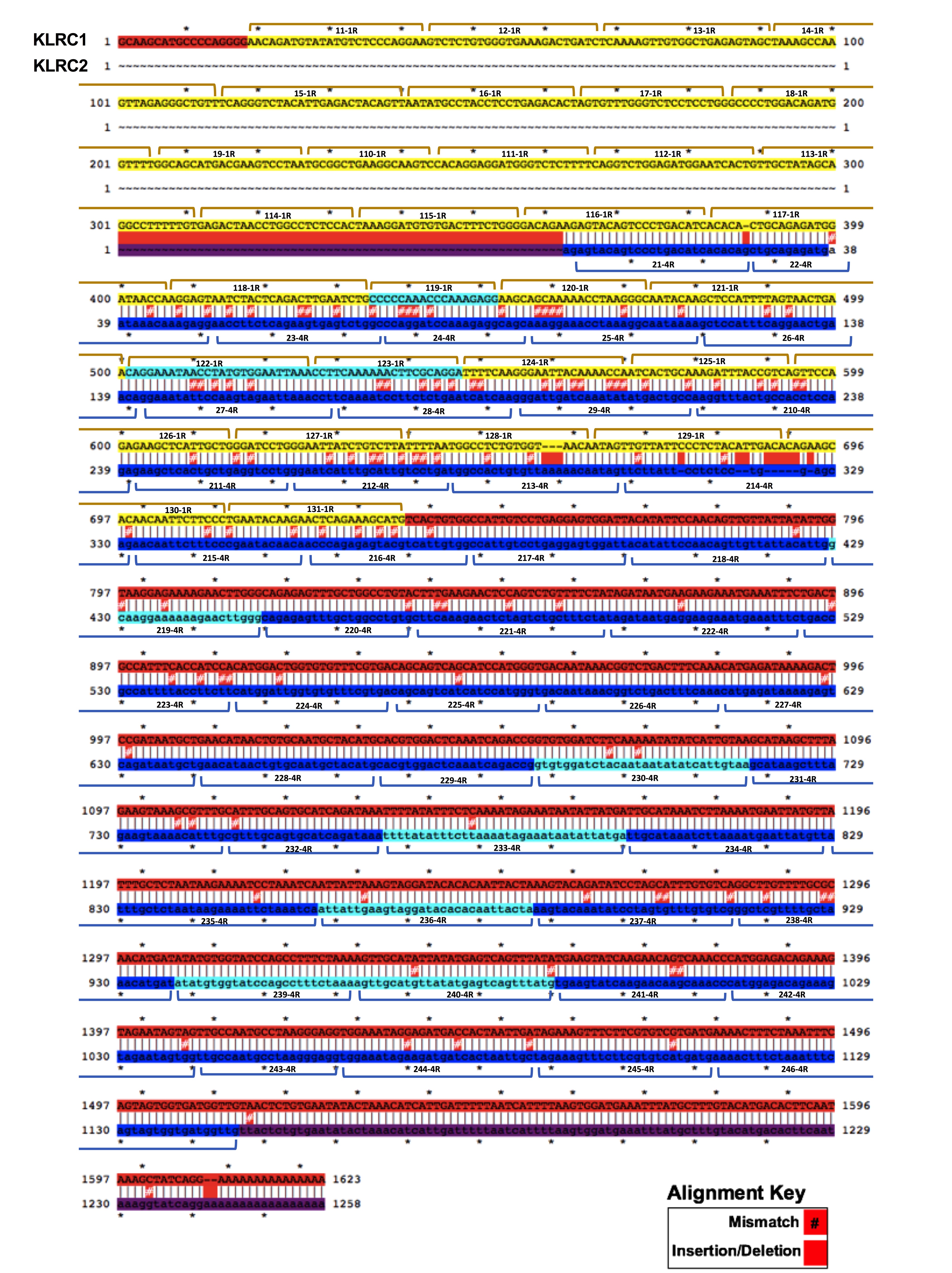

Supplement: S2 Fig — Both genes are aligned to show the sequence-specific location of all probes specific for KLRC1 and KLRC2 of all probes in S1 Fig. KLRC1 gene (red) and KLRC2 gene (purple) genes; KLRC1 probeset (blue) and KLRC2 probeset (yellow); Blocking probes (cyan). (JPG) [file ppat.1007104.s002.jpg]

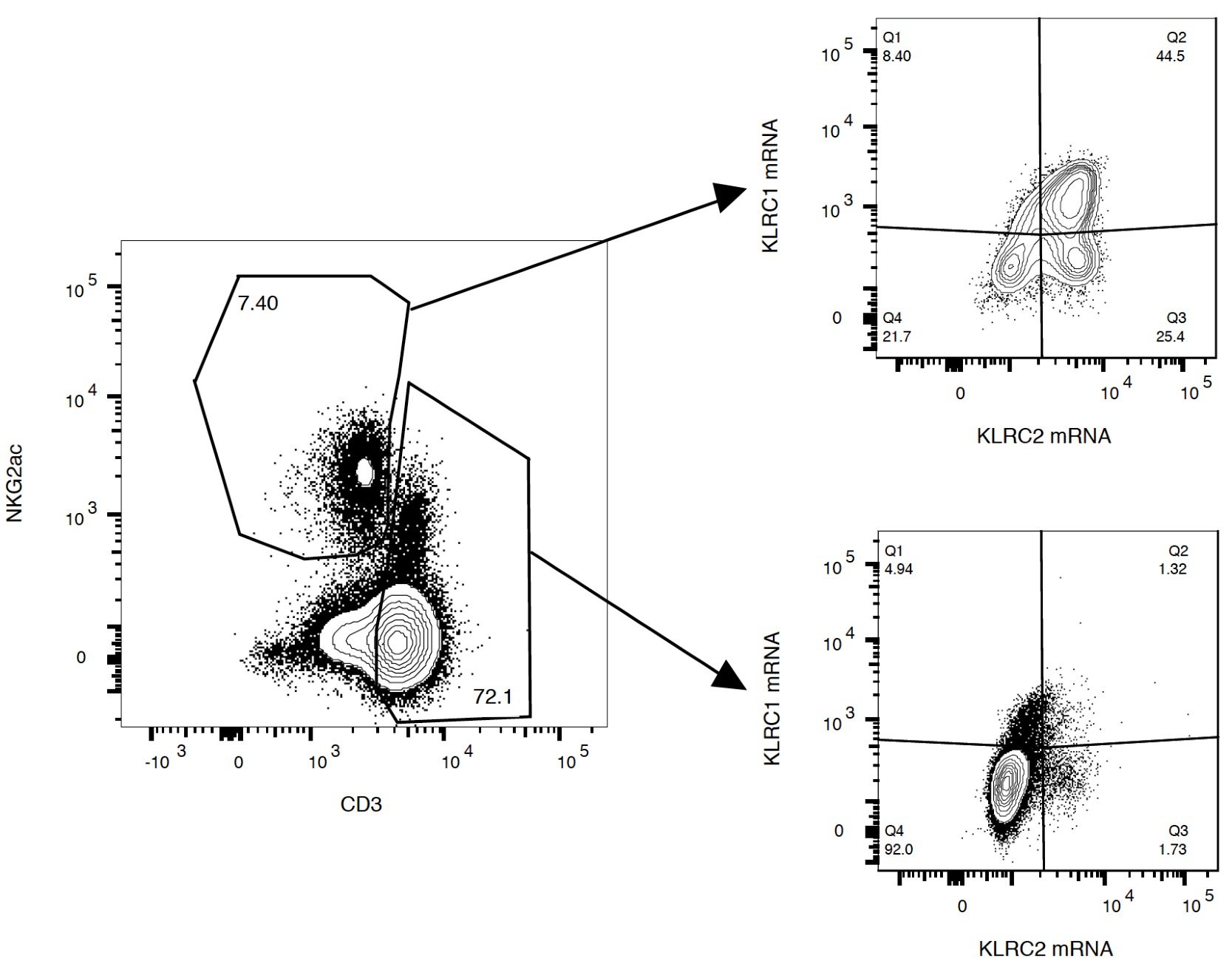

Supplement: S3 Fig — Representative plots showing expression of KLRC1 and KLRC2 on NK cells (CD14-CD20-CD3-NKG2ac+) and CD3+ T cells (CD14-CD20-CD3+NKG2ac±). (TIF) [file ppat.1007104.s003.tif]

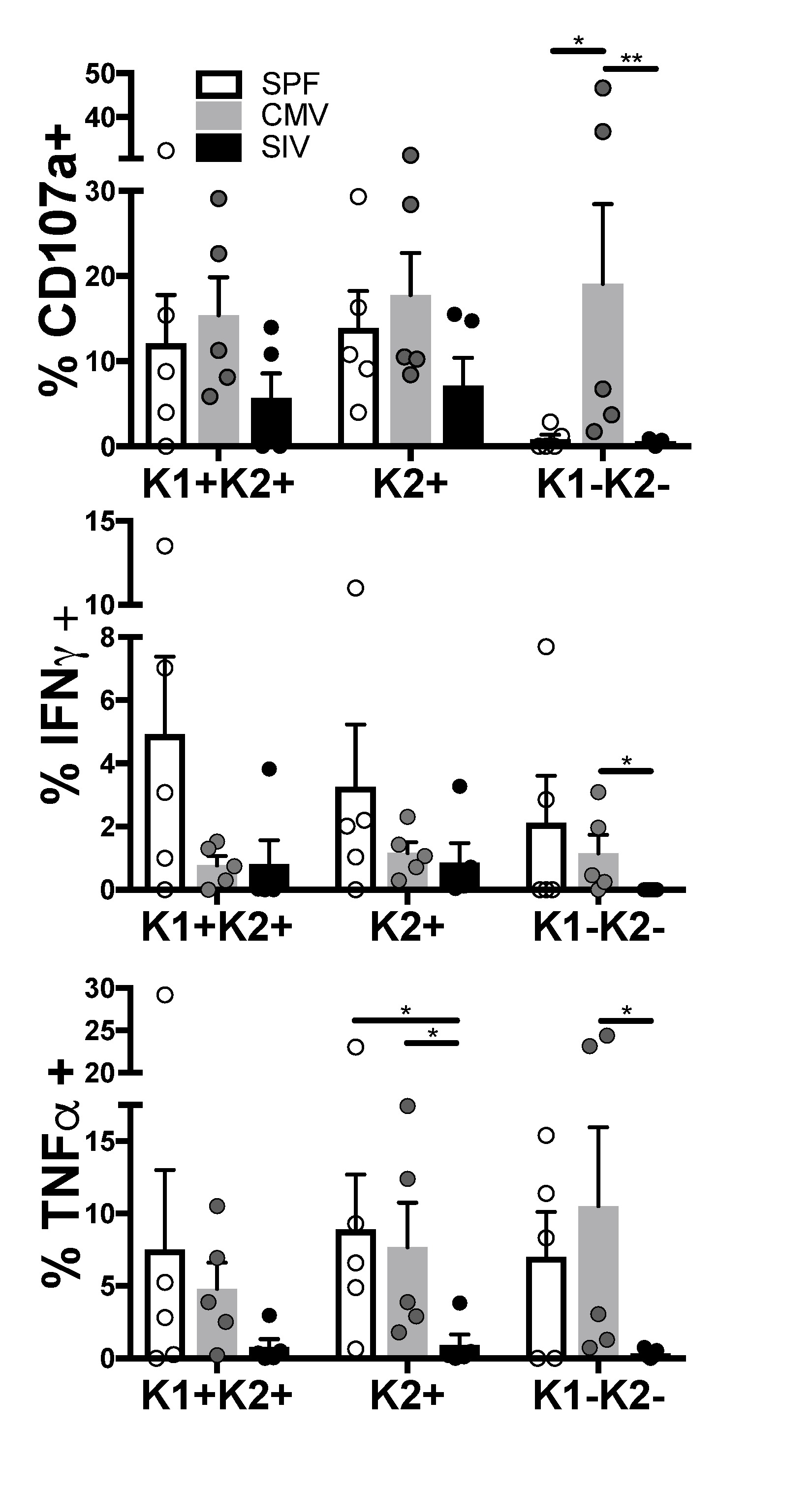

Supplement: S4 Fig — Data showing CD107a expression, or production of IFN-γ and TNF-α following stimulation with anti-CD16 cross-linked with F(ab’)2 in NK cell subpopulations from SPF, rhCMV+ or SIV+ animals. Means + SEM are shown. Numbers of animals per independent experiment: SPF (n = 5), CMV (n = 5) and SIV (n = 5). Mann-Whitney U; *p < 0.05, **p < 0.01, ***p < 0.0001. (TIF) [file ppat.1007104.s004.tif]

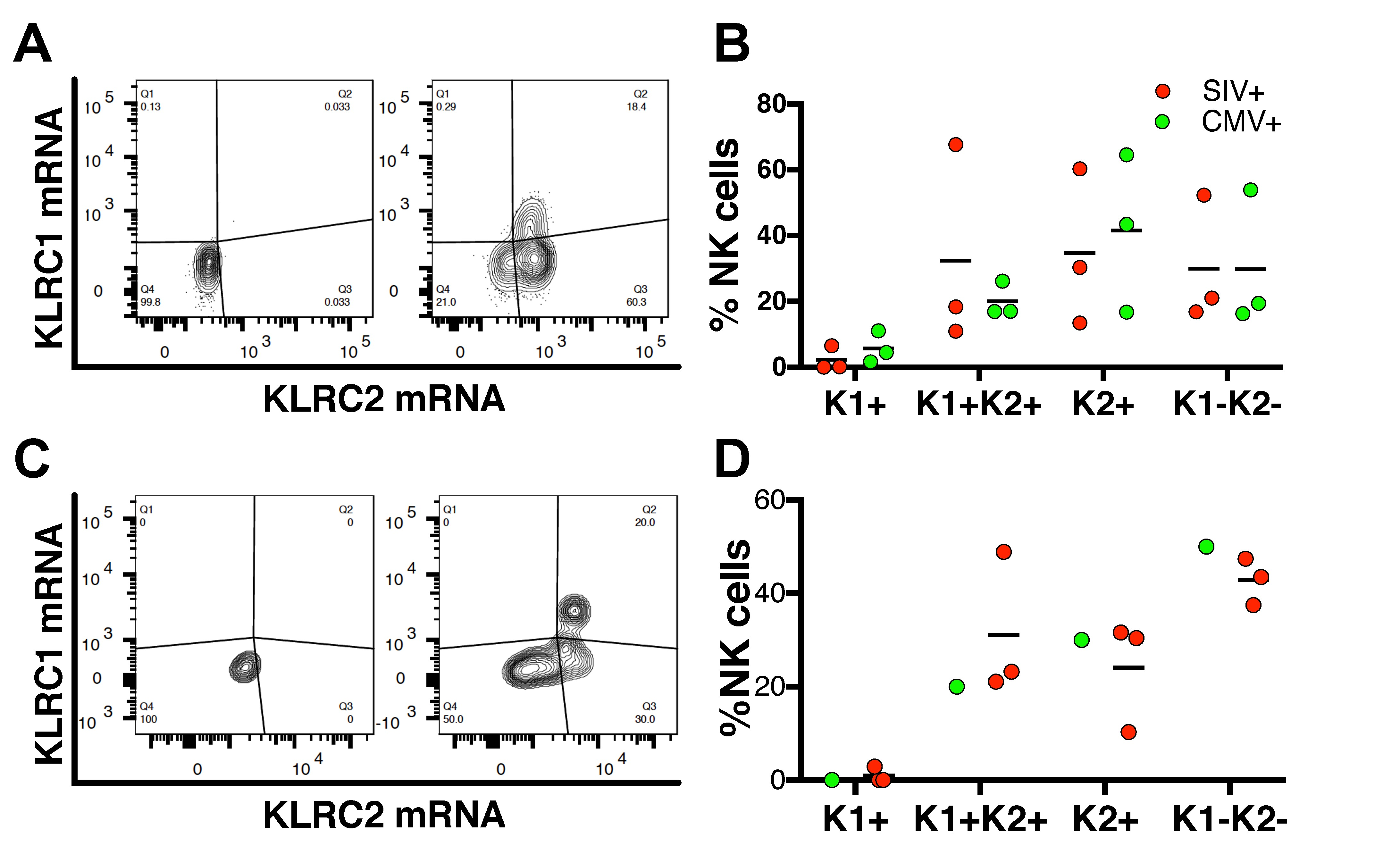

Supplement: S5 Fig — Representative flow plots showing KLRC1±KLRC2± quadrant populations in (A) Spleen and (C) Colon, as well as a distribution of NK cell KLRC1±KLRC2± subpopulations in CMV and SIV infected animals in (B) Spleen and (D) Colon. (TIF) [file ppat.1007104.s005.tif]
