# Supplementary material for: Tracking KLRC2 (NKG2C)+ memory-like NK cells in SIV+ and rhCMV+ rhesus macaques
Source: PLoS Pathog. 2018 May 31;14(5):e1007104. doi: 10.1371/journal.ppat.1007104 (PMC5997355; doi:10.1371/journal.ppat.1007104)
Supplement: S1 Table — Multiple comparisons carried out in the phenotypic assays from Fig 2C. Quadrants are represented as K2+ (KLRC1-KLRC2+), K1+K2+ (KLRC1+KLRC2+) and K1-K2- (KLRC1-KLRC2-). Shaded cells indicate comparison events that were deemed significant at p < 0.05. Non-parametric Wilcoxon test was used for inter-quadrant comparisons, and the non-parametric Mann-Whitney U test was used for inter-infection group comparisons. (DOCX) [file ppat.1007104.s006.docx]

**Supplementary Table 1**

| **Group** | Quadrants | CD16 | CD56 | KIR3D | CD2 |
| --- | --- | --- | --- | --- | --- |
| **SPF** | K1+K2+ v. K2+ | 0.0137 | 0.0039 | 0.0625 | 0.0059 |
|  | K1+K2+ v. K1-K2- | 0.0039 | 0.1035 | 0.8125 | 0.002 |
|  | K2+ v. K1-K2- | 0.002 | 0.375 | 0.0625 | 0.0273 |
|  |  |  |  |  |  |
| **CMV+** | K1+K2+ v. K2+ | 0.0005 | 0.0005 | 0.0049 | 0.0068 |
|  | K1+K2+ v. K1-K2- | 0.0005 | 0.0005 | 0.0771 | 0.0005 |
|  | K2+ v. K1-K2- | 0.0005 | 0.3101 | 0.0005 | 0.001 |
|  |  |  |  |  |  |
| **SIV+** | K1+K2+ v. K2+ | 0.0078 | 0.1094 | 0.0312 | 0.0078 |
|  | K1+K2+ v. K1-K2- | 0.0078 | 0.0547 | 0.5781 | 0.0078 |
|  | K2+ v. K1-K2- | 0.0078 | 0.0312 | 0.0312 | 0.0078 |
|  |  |  |  |  |  |
| **Quadrants** | Group |  |  |  |  |
| **K1+K2+** | SPF v. CMV | 0.2906 | 0.0011 | 0.7388 | 0.8212 |
|  | SPF v. SIV | 0.9654 | 0.0252 | 0.4318 | 0.012 |
|  | CMV v. SIV | 0.2083 | 0.3431 | 0.7108 | 0.0055 |
|  |  |  |  |  |  |
| **K2+** | SPF v. CMV | 0.03 | 0.002 | 0.6461 | 0.0138 |
|  | SPF v. SIV | 0.3154 | 0.0418 | >0.9999 | 0.0014 |
|  | CMV v. SIV | 0.0979 | 0.3431 | 0.8369 | 0.0691 |
|  |  |  |  |  |  |
| **K1-K2-** | SPF v. CMV | 0.2024 | 0.2881 | 0.3169 | 0.9229 |
|  | SPF v. SIV | 0.371 | 0.2796 | 0.3232 | 0.3154 |
|  | CMV v. SIV | 0.7345 | 0.4605 | 0.9839 | 0.1813 |

**Supplementary Table 1 NK cell phenotypic *p*-values.** Multiple comparisons carried out in the phenotypic assays from **Fig. 2C.** Quadrants are represented as K2+ (KLRC1-KLRC2+), K1+K2+ (KLRC1+KLRC2+) and K1-K2- (KLRC1-KLRC2-). Shaded cells indicate comparison events that were deemed significant at *p* < 0.05. Non-parametric Wilcoxon test was used for inter-quadrant comparisons, and the non-parametric Mann-Whitney *U* test was used for inter-infection group comparisons.
